# Supplementary material for: Multi‐dataset identification of innovative feature genes and molecular mechanisms in keratoconus
Source: J Cell Mol Med. 2024 Sep 19;28(18):e70079. doi: 10.1111/jcmm.70079 (PMC11412914; doi:10.1111/jcmm.70079)
Supplement: Supplementary file 2 — Material S2. [file JCMM-28-e70079-s002.zip › 7.GSEA analysis/5.RNF217-AS1/KEGG_TYROSINE_METABOLISM.html]

Details for gene set KEGG\_TYROSINE\_METABOLISM[GSEA]

|  || Dataset | RNF217-AS1.group.cls#h\_versus\_l |
| Phenotype | group.cls#h\_versus\_l |
| Upregulated in class | l |
| GeneSet | KEGG\_TYROSINE\_METABOLISM |
| Enrichment Score (ES) | -0.39585158 |
| Normalized Enrichment Score (NES) | -1.2715124 |
| Nominal p-value | 0.11133201 |
| FDR q-value | 0.41937515 |
| FWER p-Value | 0.966 |
Table: GSEA Results Summary

  

Fig 1: Enrichment plot: KEGG\_TYROSINE\_METABOLISM      
 Profile of the Running ES Score & Positions of GeneSet Members on the Rank Ordered List

  

| SYMBOL | TITLE | RANK IN GENE LIST | RANK METRIC SCORE | RUNNING ES | CORE ENRICHMENT || 1 | IL4I1 | na | 570 | 0.516 | 0.0493 | No |
| 2 | TAT | na | 1456 | 0.397 | 0.0745 | No |
| 3 | MAOB | na | 1741 | 0.370 | 0.1135 | No |
| 4 | ADH1B | na | 3511 | 0.271 | 0.0971 | No |
| 5 | ADH6 | na | 4165 | 0.247 | 0.1097 | No |
| 6 | ADH4 | na | 4223 | 0.244 | 0.1392 | No |
| 7 | AOC2 | na | 5673 | 0.193 | 0.1221 | No |
| 8 | TH | na | 7858 | 0.131 | 0.0758 | No |
| 9 | TRMT11 | na | 10210 | 0.083 | 0.0186 | No |
| 10 | TPO | na | 15177 | 0.017 | -0.1223 | No |
| 11 | METTL2B | na | 15371 | 0.015 | -0.1259 | No |
| 12 | GSTZ1 | na | 15858 | 0.010 | -0.1386 | No |
| 13 | DDC | na | 16555 | 0.003 | -0.1583 | No |
| 14 | ADH7 | na | 16853 | 0.000 | -0.1669 | No |
| 15 | LCMT1 | na | 17666 | -0.008 | -0.1893 | No |
| 16 | GOT2 | na | 17814 | -0.009 | -0.1923 | No |
| 17 | MIF | na | 18730 | -0.018 | -0.2165 | No |
| 18 | MAOA | na | 19080 | -0.021 | -0.2239 | No |
| 19 | FAH | na | 19343 | -0.024 | -0.2284 | No |
| 20 | GOT1 | na | 20120 | -0.031 | -0.2468 | No |
| 21 | ALDH3B2 | na | 23124 | -0.063 | -0.3253 | No |
| 22 | COMT | na | 23764 | -0.072 | -0.3346 | No |
| 23 | ALDH3A1 | na | 23827 | -0.073 | -0.3271 | No |
| 24 | ALDH3B1 | na | 24098 | -0.077 | -0.3251 | No |
| 25 | LCMT2 | na | 24680 | -0.086 | -0.3310 | No |
| 26 | ADH1A | na | 24873 | -0.088 | -0.3252 | No |
| 27 | METTL6 | na | 26062 | -0.108 | -0.3457 | No |
| 28 | ALDH1A3 | na | 26655 | -0.119 | -0.3476 | No |
| 29 | HEMK1 | na | 27571 | -0.138 | -0.3563 | Yes |
| 30 | AOX1 | na | 27971 | -0.148 | -0.3489 | Yes |
| 31 | HPD | na | 27994 | -0.149 | -0.3306 | Yes |
| 32 | AOC3 | na | 28131 | -0.152 | -0.3152 | Yes |
| 33 | ADH5 | na | 28827 | -0.171 | -0.3133 | Yes |
| 34 | HGD | na | 30815 | -0.234 | -0.3407 | Yes |
| 35 | DCT | na | 32728 | -0.339 | -0.3526 | Yes |
| 36 | PNMT | na | 33209 | -0.377 | -0.3184 | Yes |
| 37 | DBH | na | 33634 | -0.424 | -0.2766 | Yes |
| 38 | ADH1C | na | 33785 | -0.445 | -0.2241 | Yes |
| 39 | TYRP1 | na | 34684 | -0.897 | -0.1356 | Yes |
| 40 | TYR | na | 34713 | -1.073 | 0.0004 | Yes |
Table: GSEA details [plain text format]

  

Fig 2: KEGG\_TYROSINE\_METABOLISM      
 Blue-Pink O' Gram in the Space of the Analyzed GeneSet

  

Fig 3: KEGG\_TYROSINE\_METABOLISM: Random ES distribution      
 Gene set null distribution of ES for **KEGG\_TYROSINE\_METABOLISM**
